# A Novel Defined Risk Signature of the Ferroptosis-Related Genes for Predicting the Prognosis of Ovarian Cancer

**DOI:** 10.3389/fmolb.2021.645845

**Published:** 2021-04-01

**Authors:** Ying Ye, Qinjin Dai, Shuhong Li, Jie He, Hongbo Qi

**Affiliations:** ^1^The Department of Obstetrics, The First Affiliated Hospital of Chongqing Medical University, Chongqing, China; ^2^State Key Laboratory of Maternal and Fetal Medicine of Chongqing Municipality, Chongqing Medical University, Chongqing, China; ^3^Guangzhou Women and Children's Medical Center, Guangzhou Medical University, Guangzhou, Guangdong, China; ^4^The Department of Oncology, The First Affiliated Hospital of Chongqing Medical University, Chongqing, China

**Keywords:** ovarian cancer, ferroptosis, risk signature, overall survival, immune cell infiltration

## Abstract

Ferroptosis is an iron-dependent, regulated form of cell death, and the process is complex, consisting of a variety of metabolites and biological molecules. Ovarian cancer (OC) is a highly malignant gynecologic tumor with a poor survival rate. However, the predictive role of ferroptosis-related genes in ovarian cancer prognosis remains unknown. In this study, we demonstrated that the 57 ferroptosis-related genes were expressed differently between ovarian cancer and normal ovarian tissue, and based on these genes, all OC cases can be well divided into 2 subgroups by applying consensus clustering. We utilized the least absolute shrinkage and selection operator (LASSO) cox regression model to develop a multigene risk signature from the TCGA cohort and then validated it in an OC cohort from the GEO database. A 5-gene signature was built and reveals a favorable predictive efficacy in both TCGA and GEO cohort (*P* < 0.001 and *P* = 0.03). The GO and KEGG analysis revealed that the differentially expressed genes (DEGs) between the low- and high-risk subgroup divided by our risk model were associated with tumor immunity, and lower immune status in the high-risk group was discovered. In conclusion, ferroptosis-related genes are vital factors predicting the prognosis of OC and could be a novel potential treatment target.

## Introduction

Ovarian cancer (OC) is the most lethal malignancy among gynecological tumors and causes ~150,000 women to death every year (Lheureux et al., 2019). Due to the lack of typical clinical symptoms in the early phases, 75% of OC patients are diagnosed at advanced stages, and more than 70% of patients experience recurrence after treatment (Matulonis et al., 2016). The therapeutic drugs against OC were quickly progressed in the past 20 years, however, the overall survival (OS) was still poorly increased in most countries (Lee et al., 2018). As the current treatment measures are not promising, identifying reliable prognostic biomarkers is important to prolong the survival time of OC patients. In clinical practice, CA-125 and Human epididymis protein 4 (*HE4*) were the most commonly used predictive markers (Piatek et al., 2020; Salminen et al., 2020). Besides, recent studies indicated that some microRNAs (miRNAs) and long non-coding RNAs (lncRNAs) were also associated with the prognosis of OC (Nam et al., 2008; Qiu et al., 2014). However, as the molecular mechanisms affecting the prognosis of ovarian cancer are complex, single gene/factor prediction models are often with low accuracy. In contrast, multiple-gene-based models often showed better efficacies in predicting the prognosis of various tumors.

Ferroptosis is an iron-dependent, non-apoptotic form of the regulated cell death process that is regulated by a variety of genes (Chen et al., 2021). It presented unique morphological characteristics, such as reduced mitochondrial volume, increased mitochondrial membrane density, and reduced or absent mitochondrial cristae, but with intact cell membranes, normal nucleus size, and no chromatin condensation (Dixon et al., 2012). Biochemical features of ferroptosis are characterized by elevated levels of iron ions, production of large amounts of reactive oxygen species decreased activity of glutathione peroxidase 4 (*GPx4*), and accumulation of lipid metabolites (Yang and Stockwell, 2016). The occurrence of ferroptosis is closely related to cysteine metabolism, lipid metabolism, and iron cycle: inhibition of cysteine production and reduced glutathione are key components of iron death; polyunsaturated fatty acids are prone to lipid peroxidation that directly induces iron death; iron ion uptake, elimination, storage, and content can influence the sensitivity of cells to iron death (Xie et al., 2016; Conrad et al., 2018). Ferroptosis is associated with a variety of diseases, including neurological disorders, ischemia/reperfusion injury, kidney damage, and blood disorders (Li et al., 2020b). Many studies suggested that ferroptosis is closely linked to the regulation of growth in a variety of tumor cells, including OC. Iron metabolism is potentially related to ovarian cancer cell growth and metastasis. Compared to normal ovarian tissues, OC tissues always showed decreased ferroportin (*FPN*), increased transferrin receptor-1 (*TFR1*), and transferrin (*TF*), and resulted in elevated iron levels (Basuli et al., 2017). In *in vitro*, the proliferation of OC cells was observed to be inhibited by increased iron content, while in animal models, the increasing iron concentration showed inhibition of tumor growth and decreased intraperitoneal dissemination (Basuli et al., 2017). This suggests that disrupting iron metabolism is a potential target for killing OC cells.

Combined with the existing findings, we know ferroptosis plays important role in the development of tumors and anti-tumor procedures, however, its specific functions in ovarian cancer have not been fully elucidated. Therefore, exploring the relationships between ferroptosis and OC is essential to our understanding of the mechanisms of tumor development, and constructing a predictive model based on ferroptosis-related genes is of great clinical significance for prolonging the survival time of OC patients. Moreover, current chemotherapy regimens for ovarian cancer are still dominated by platinum and paclitaxel drugs, but the prognosis for patients with advanced ovarian cancer remains bleak. Accordingly, an in-depth investigation of the ferroptosis pathway and ovarian cancer will provide strategies for developing novel targeted regimens.

For this purpose, we performed a study combining the GTEx and the TCGA databases to make a comprehensive understanding of the expression levels of the ferroptosis-related genes between normal ovarian and OC tissues. We further screened the genes which were closely connected to the survival rate of OC patients and constructed a risk signature based on 5 ferroptosis-related genes. By employing an external GEO cohort, we validated the model's accuracy and reliability. Moreover, combined with clinical features, we found the gene signature was an independent predictive factor for OC patients. Overall, our study successfully conducted a ferroptosis-related risk model and can potentially be applied for clinical treatment and diagnosis.

## Materials and Methods

### Data Resources

The mRNA expression profiles and clinical information of 379 OC patients were downloaded from The Cancer Genome Atlas (TCGA) (https://portal.gdc.cancer.gov/). Eighty eight normal ovarian samples with RNA sequencing data were got from the Genotype-Tissue Expression (GTEx) database (https://xenabrowser.net/). Besides, the external validation data of 415 OC patients (with RNA-seq and clinical data) was obtained from the Gene Expression Omnibus (GEO) database (https://www.ncbi.nlm.nih.gov/geo/; GSE13876). The expression data in each database was firstly normalized to fragment per kilobase million (FPKM) values. Before further comparison, the “scale” function in the “limma” R package (version 4.0.3) was applied to normalize data among databases. We gathered 62 ferroptosis-related genes from prior studies and were presented in Supplementary table 1 (Dixon et al., 2012; Stockwell et al., 2017; Zheng and Conrad, 2020).

### Identification of Differentially Expressed Ferroptosis-Related Genes

Firstly, we combined the TCGA (379 OC tissues) and the GTEx (88 normal ovarian tissues) database to identify the differentially expressed genes (DEGs, with false discovery rate (FDR) < 0.05 and |log_2_ FC|≥ 0) from 62 ferroptosis regulators. The heatmap reflecting the expression levels of each DEG between normal and tumor tissues was performed by the “heatmap” R package. To better know the connections among DEGs, a protein-protein interactions (PPI) analysis was conducted by using the Search Tool for the Retrieval of Interacting Genes (STRING) online tool (http://string-db.org/). To explore the connections between the expression of these ferroptosis-related DEGs and OC subtypes, we employed the consensus clustering analysis (utilizing the “limma” R package) to realize the tumor classification in the TCGA cohort. The principal component analysis (PCA) conducted by the “ggplot2” R package was utilized to verify the accuracy of classifications. The Kaplan–Meier survival curve analysis was utilized to compare the survival possibility among tumor subtypes. Moreover, the clinicopathologic characters (tumor grade and age) were also compared in different subtypes.

### Construction and Validation of a Ferroptosis-Related Gene Signature

We next screened out the survival-related genes using univariate cox analysis for further developing a risk signature with the least absolute shrinkage and selection operator (LASSO) cox regression model (By employing the “glmnet” and “survival” R packages). The risk score was calculated by the formula: Risk scores= $\overset{n}{\underset{i}{\sum }}Xi\times Yi$ (X: coefficient of each gene, Y: expression of each gene). Based on the median score, OC patients from the TCGA database were divided into low- and high-risk subgroups. The OS time was compared between the two groups using the Kaplan–Meier survival curve analysis and the ROC curve was used for evaluating the sensitivity and specificity of the gene signature. To validate our model, we obtained the data from a cohort (GEO database, GSE13876). The risk score of each patient in the GEO cohort was calculated by the same formula applied in the TCGA cohort. According to the risk score, patients in the GEO cohort were also divided into low- and high-risk subgroups, and the OS was then compared between the two risk subgroups.

### Functional Enrichment Analysis

**Figure 1:**
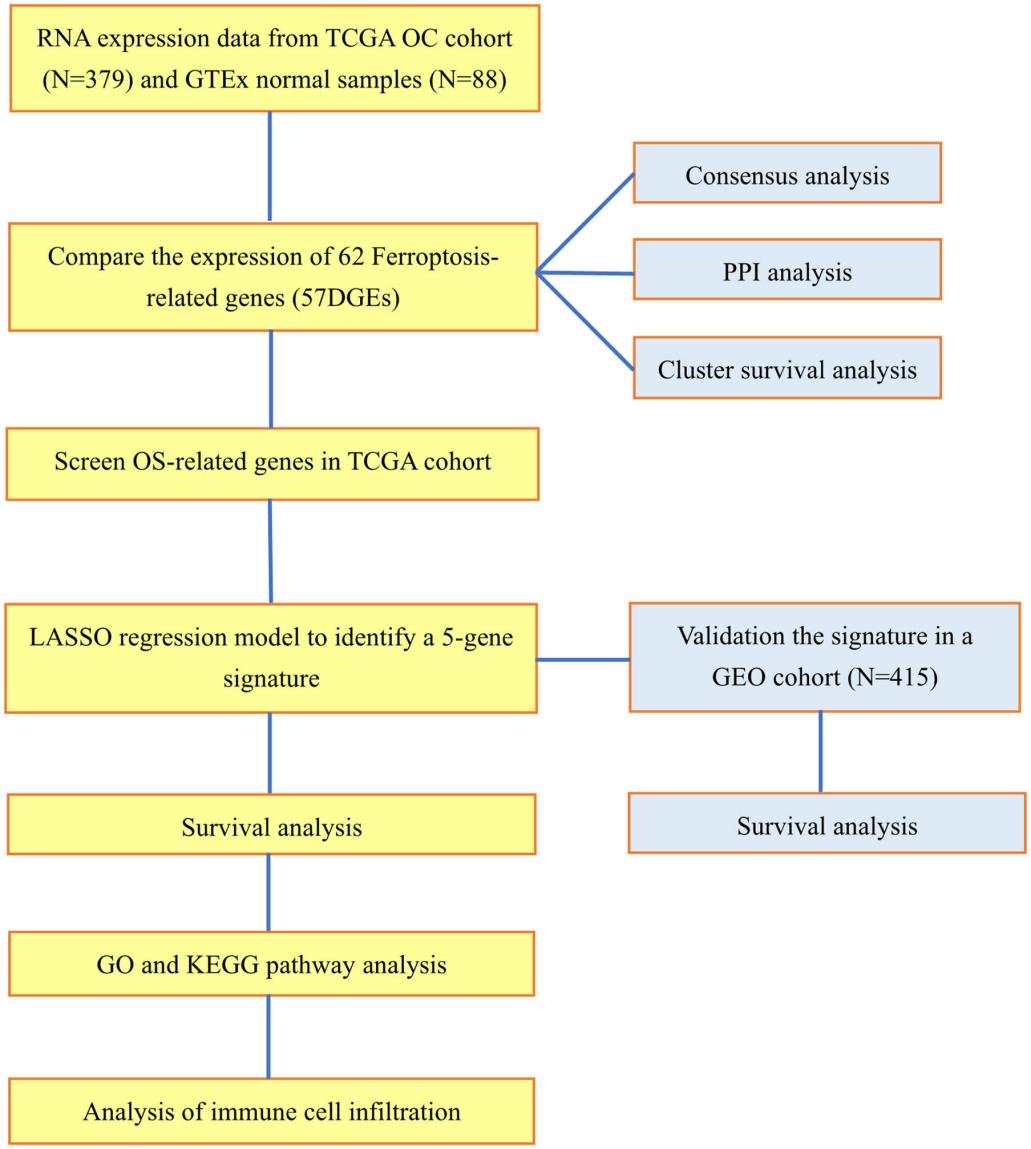
Workflow diagraph of data collection and analysis.

In the TCGA cohort, 379 OC patients were classified into 2 risk groups according to the risk scores based on the ferroptosis signature. The differentially expressed genes between the 2 groups were then screened out by the "limma” package using the criteria: FDR <0.05 and |log_2_FC| ≥ 1. We then applied the “limma” and the “clusterProfiler” packages to perform the Gene Ontology (GO) analysis and Kyoto Encyclopedia of Genes and Genomes (KEGG) pathway enrichment analysis. As for the GO and KEGG results that the diverse genes were connected with the aggregation of immune cells, thus we applied the single-sample gene set enrichment analysis (ssGSEA) to assess the infiltration scores of immune cells and the activity of immune-related functions. The molecular markers of each immune cell and pathway were presented in Supplementary Table 2. The workflow diagram is shown in Figure 1.

### Statistical Analysis

The *t*-test was used to contrast the expression levels of genes between normal ovarian and OC tissues, while the Pearson chi-square test was employed to compare the categorical variables. The Kaplan-Meier curve with a two-sided log-rank test was applied to compare the OS of patients between subgroups. Univariate and multivariate Cox regression analyses were conducted to figure out the independent factors related to survival rate. The Mann-Whitney test was used to compare the scores of infiltrating immune cells and the activities of immune-related pathways between low- and high-risk groups. All statistical analyses were executed utilizing R v4.0.2.

## Results

### Tumor Classification Based on the Differential Expression of Ferroptosis-Related Genes

**Figure 2:**
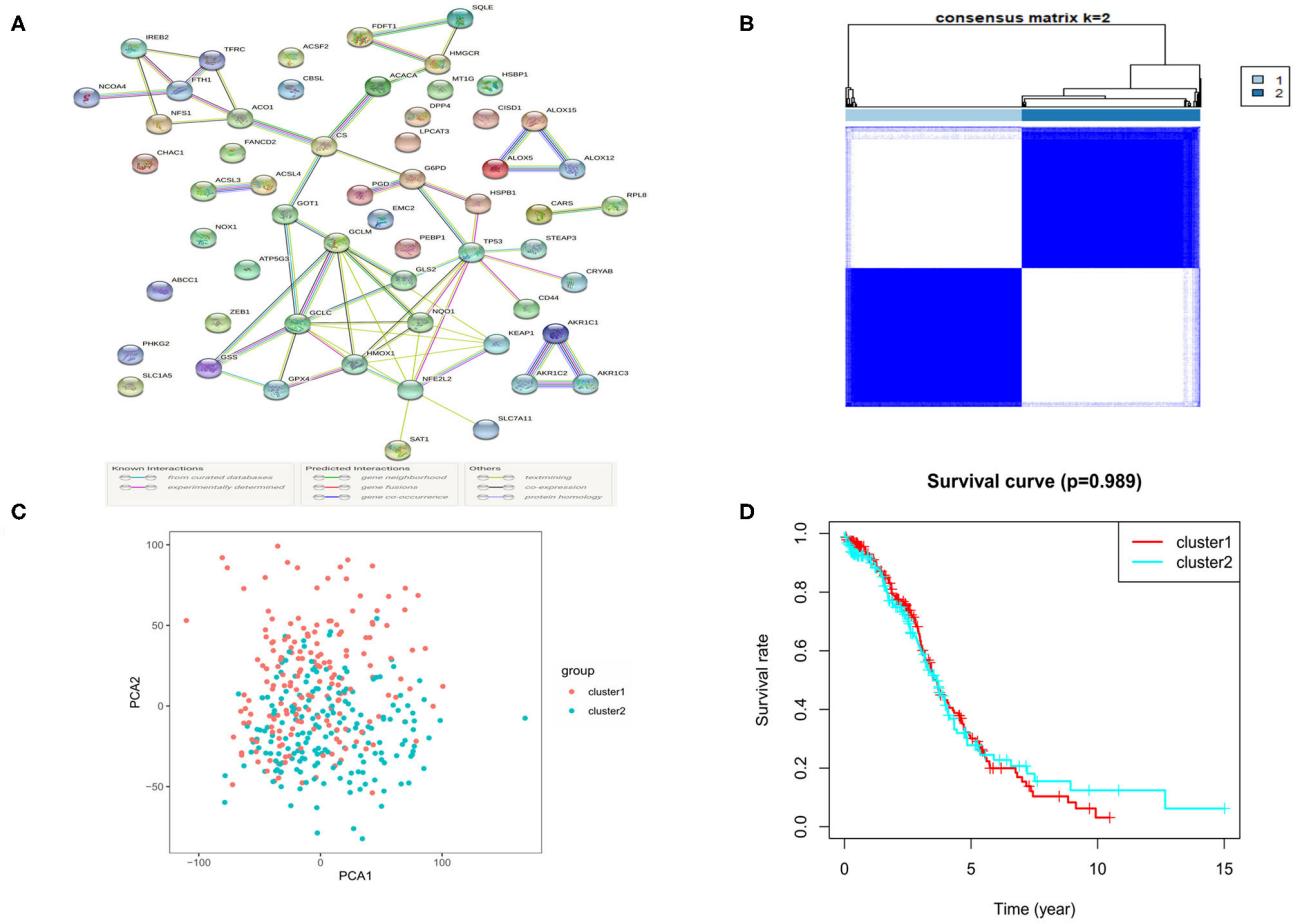
The Consensus clustering for 379 OC samples based on the DEGs. **(A)** PPI network showing the interactions of DEGs. **(B)** Consensus clustering matrix for k = 2. **(C)** PCA plot for the consensus clustering matrix. **(D)** Kaplan-Meier OS curves for the OC patients in the 2 clusters.

The RNA-seq data of 88 normal ovarian tissues from the GTEx database and 379 OC tissues from the TCGA cohort were merged and normalized to make further comparisons. The expression levels of 62 ferroptosis-related genes which were extracted from prior review articles were compared. In the pooled dataset, we identified 57 genes were with significantly different expressions (*P* < 0.05). Among them, 26 were down-regulated while the other 31 were up-regulated in tumor samples and the heatmaps for these DEGs were presented in Supplementary Figure 1. A PPI analysis was performed to show the relationships among the 57 DEGs, and the minimum required interaction score was set at 0.70 (Figure 2A).

To further evaluate the relationship between the expression profile of ferroptosis regulators and the OC subtypes, the consensus clustering analysis was performed to make tumor classifications for the 379 OC samples. We increased the clustering variable (k) from 2 to 10 and found that when k = 2, it presented a high intra-group and a low inter-group correlation, indicating that it is suitable to divide OC patients into two subtypes (cluster 1 and 2) according to the 57 ferroptosis-related DEGs (Figure 2B). A principal component analysis (PCA) based on the whole transcriptome profiles of the 2 clusters was then conducted, and an obvious separation of the clusters was observed, demonstrating a satisfactory classification of the tumor (Figure 2C). The overall survival (OS) rate was compared between the 2 clusters, but no obvious difference was found (*P* = 0.989, Figure 2D). We further compared the clinical features (tumor grade, age, and survival status) between the 2 clusters, but found few differences (Supplementary Figure 2).

### Development of a Risk Signature in the TCGA Cohort

**Figure 3:**
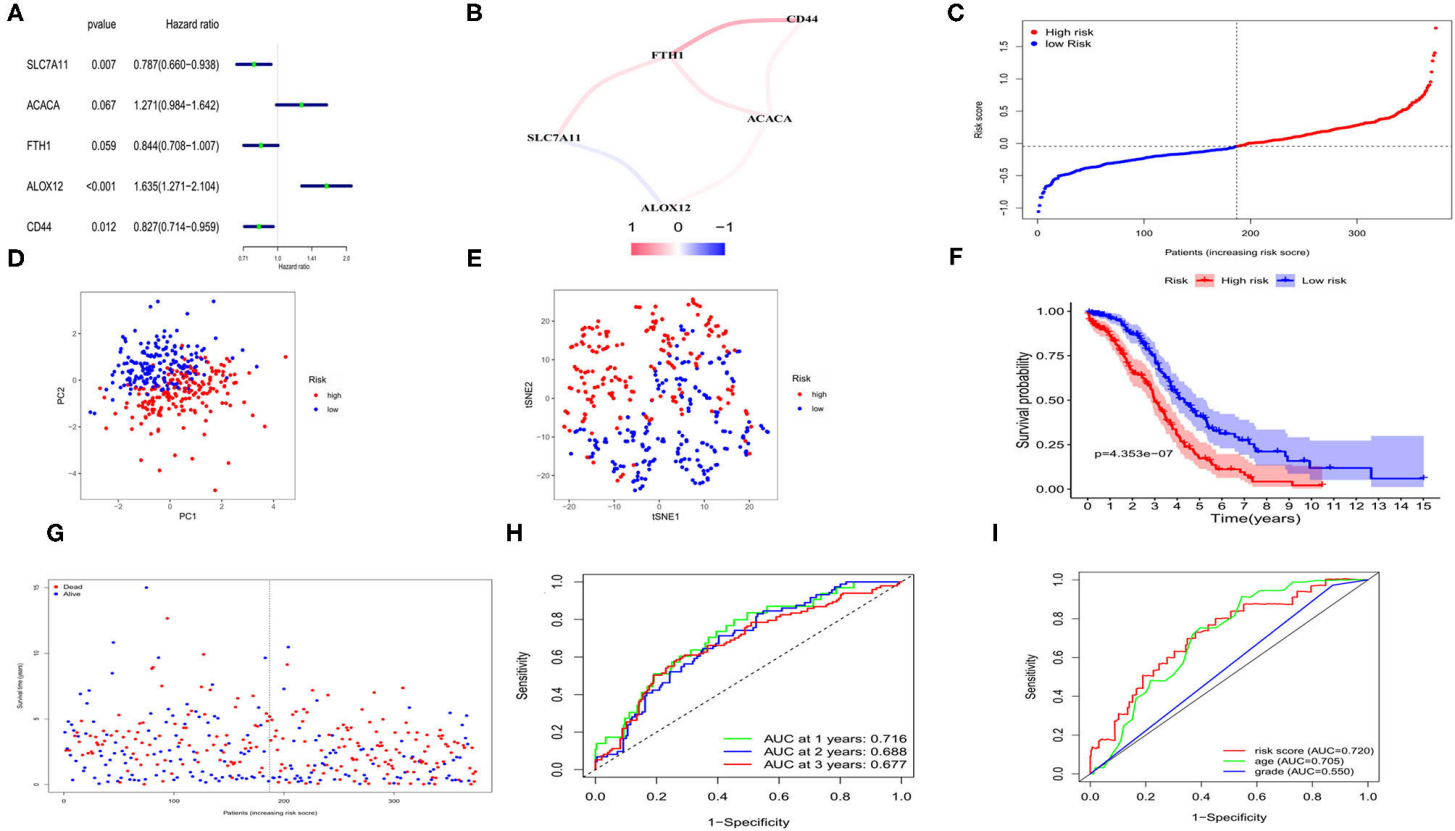
Construction of risk signature for OCs in TCGA cohort. **(A)** Screen out of the OS-related genes with univariate cox regression analysis. **(B)** The correlation network of the OS-related genes. **(C)** Risk score for OCs. **(D)** PCA plot for OCs. **(E)** t-SNE analysis for OCs. **(F)** Kaplan-Meier curves for the OS of patients in the high- and low-risk group based on risk score. **(G)** Survival status for each patient. **(H)** ROC curves demonstrated the predictive efficiency. **(I)** Multi-indicator ROC curves for risk score, age and tumor grade.

We applied univariate Cox regression analysis to evaluate the correlations between the 57 ferroptosis-related DEGs and the OS time in the TCGA cohort. Five possible survival-related genes were screened out (with *P* < 0.1). The forest plot of the 5 genes (*SLC7A11, ACACA, FTH1, ALOX12, CD44*) was shown in Figure 3A. A correlation network based on the expression profiles of the 5 genes was performed to show the relationships among them (red: positive correlations; blue: negative correlations, Figure 3B). LASSO cox regression model was then used to set up a prognostic model from the 5 genes. Finally, all of the 5 genes and their coefficients were retained, according to the penalty parameter (λ) decided by the minimum criteria (Supplementary Figure 3). The risk score was calculated for each case, and referred to the median risk score, 187 patients were classified as the low-risk group, while the other 187 were at high risk (5 cases without clinical information or with 0 days of survival time were excluded) (Figure 3C). The formula of risk score was: risk score = (0.445*ALOX12 exp) + (-0.218*SLC7A11 exp) + (-0.090*FTH1 exp) + (0.285*ACACA exp) + (-0.153*CD44 exp). The PCA and t-distributed stochastic neighbor embedding (t-SNE) analysis implied the patients with different risks were well separated in two directions (Figures 3D,E). We detected a notable difference in survival probability between the two risk subgroups (Figure 3F, *P* < 0.001). In Figure 3G, we made a visualization that patients in the high-risk group had a higher rate of death and a lower survival time than those in the low-risk group. The time-dependent receiver operating characteristic (ROC) curve was used for evaluating the predictive role of risk scores on OS and the area under the curve (AUC) was 0.716 for 1 year, 0.688 for 2 years, and 0.677 for 3 years (Figure 3H). By applying the multi-indicator ROC curve analysis, we further evaluated the predictive efficacy of the risk score and the clinical features, and the results indicated that the predictive accuracy of our risk model was superior to age and tumor grade (AUC: 0.720 for the risk model, 0.705 for age and 0.550 for tumor grade; Figure 3I).

### Validation of the Risk Signature in a GEO OC Cohort

**Figure 4:**
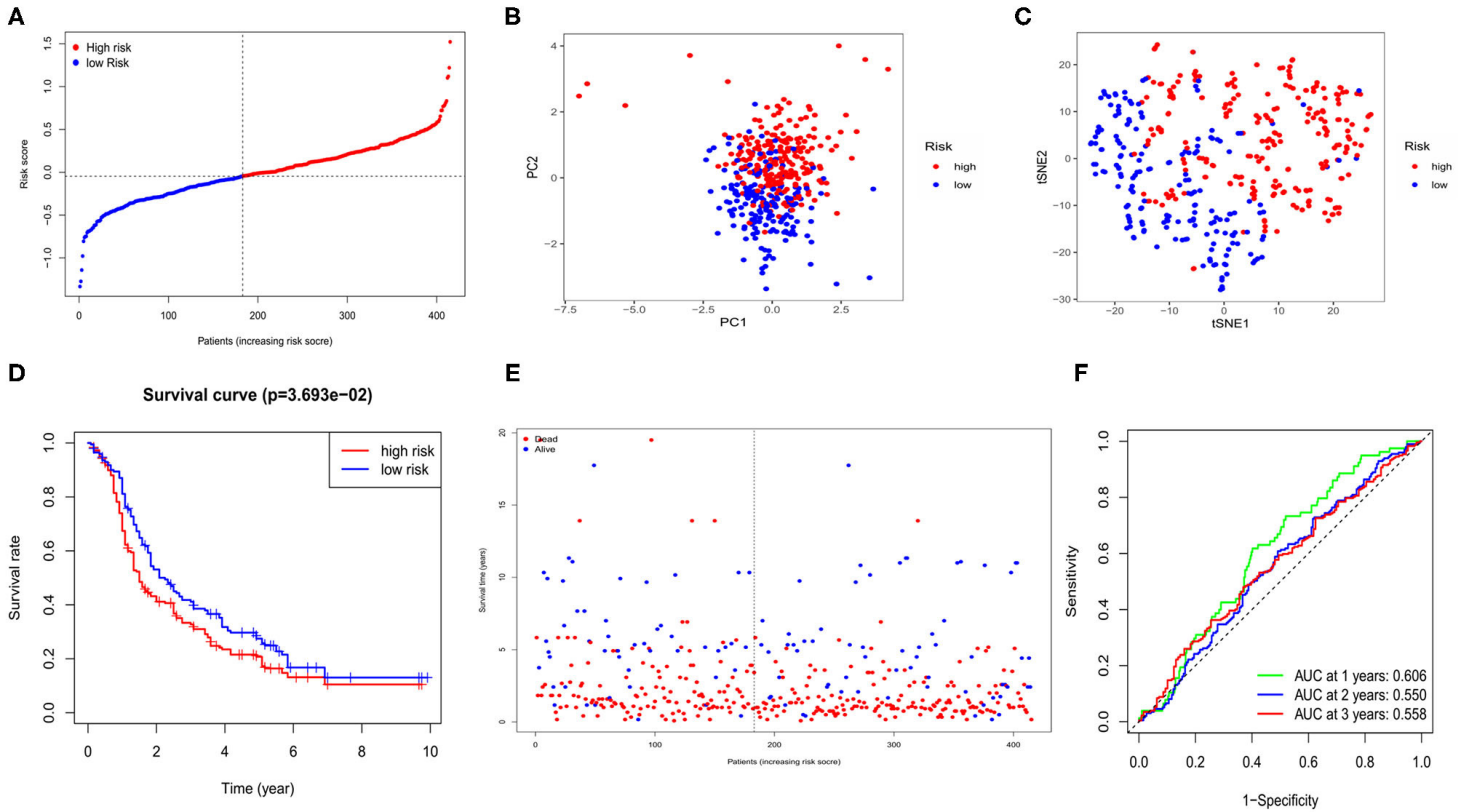
Validation of risk signature for OCs in GEO cohort. **(A)** Risk score for OCs. **(B)** PCA plot for OCs. **(C)** t-SNE analysis for OCs. **(D)** Kaplan-Meier curves for the OS of patients in GEO cohort. **(E)** The distributions of survival status. **(F)** Time-dependent ROC curves for OCs in GEO cohort.

Four hundred and fifteen OC cases with completed clinical data from the GEO database (GSE13876) were treated as the external validation set. The five survival-related genes were extracted from the expression profiling array, and before validation, the expression data of each gene from both the TCGA and the GEO databases was normalized by the “SCALE” function to avoid deviations caused by different sequencing platforms. The risk score of each case in the GEO cohort was calculated by the same formula used in the TCGA cohort. Regarding the median risk score in the TCGA cohort as the standard for the risk setting in the GEO cohort, 183 cases were allocated in the low-risk group while the other 232 were at high risk (Figure 4A). The PCA and t-SNE analysis also presented well separations between the two subgroups (Figures 4B,C). We also found that the high-risk subgroup had a significantly lower survival possibility than the low-risk group (*P* = 0.03, Figure 4D). Similarly, the survival status graph also indicated a higher possibility of earlier death in the high-risk group (Figure 4E). The time-ROC curve also revealed a passably predictive role of our model in the GEO cohort (Figure 4F).

### Independent Prognostic Value of the 5-Gene Signature

**Figure 5:**
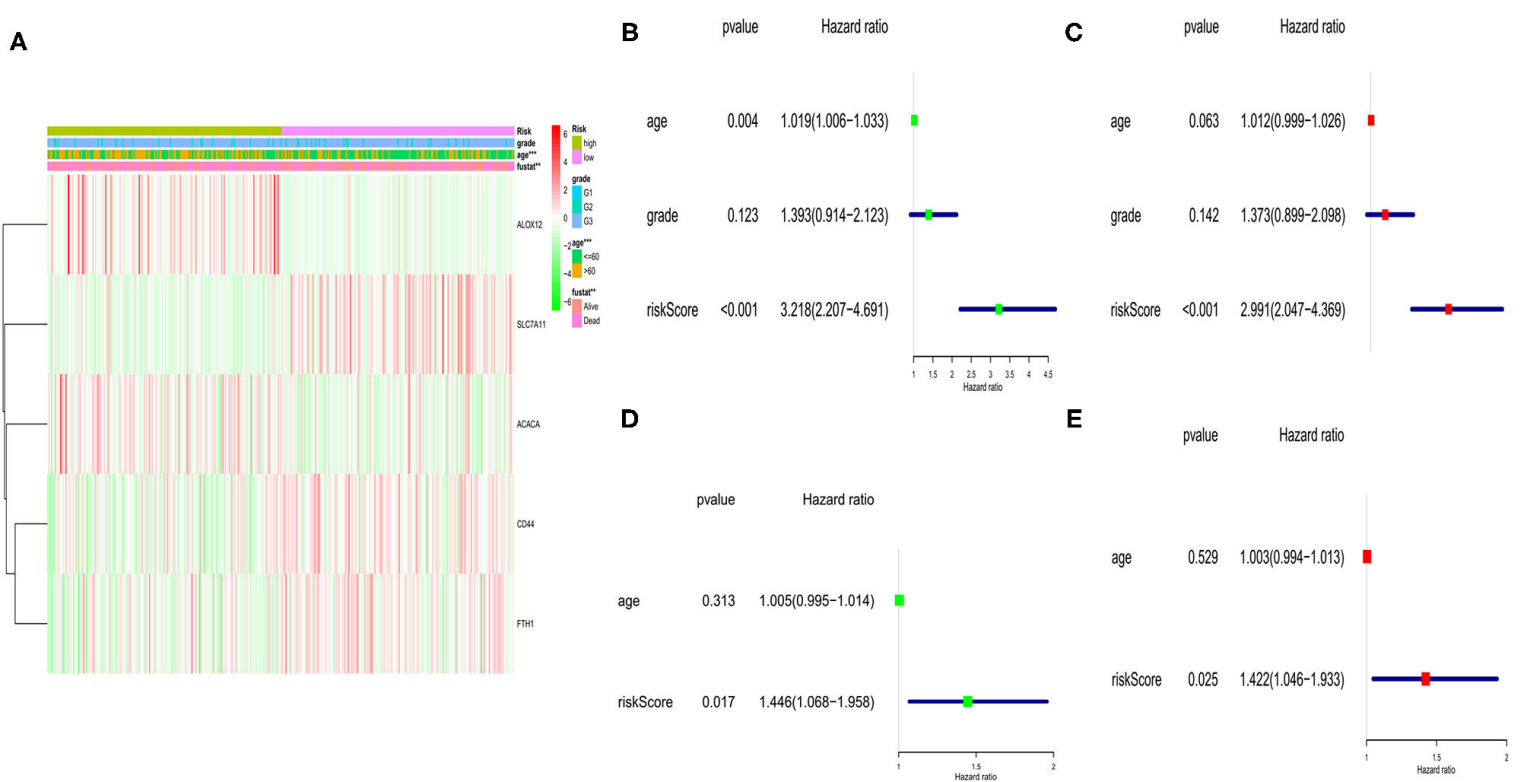
Univariate and multivariate Cox regression analyses of OS in TCGA and GEO cohort. **(A)** Heatmap and clinicopathologic features of the 5 ferroptosis-related genes. **(B)** Univariate analysis for TCGA cohort. **(C)** Multivariate analysis for TCGA cohort. **(D)** Univariate analysis for GEO cohort. **(E)** Multivariate analysis for GEO cohort.

Here, we performed a heatmap to directly show the relationships between the 5 genes and clinical features TCGA cohort (Figure 5A). Among them, 2 genes (*ALOX12* and *ACACA*) were down-regulated, while *SLC7A11, FTH1*, and *CD44* were up-regulated in the low-risk subgroup. Moreover, univariate and multivariate analyses were applied to explore whether the risk score could be an independent risk factor in both the TCGA and GEO cohorts. As shown in Figures 5B,C, the risk score was a reliable independent risk factor connected with OS (*P* < 0.001, HR:2.991, 95% CI:2.047-4.369) in TCGA cases. In the GEO cohort (lacking the tumor stage information), we also found that our risk model could be an independent risk factor of the OS (*P* = 0.025, HR:1.422, 95% CI:1.046-1.933, Figures 5D,E).

### The Exploration of Differential Gene Functions Based on the Risk Model

**Figure 6:**
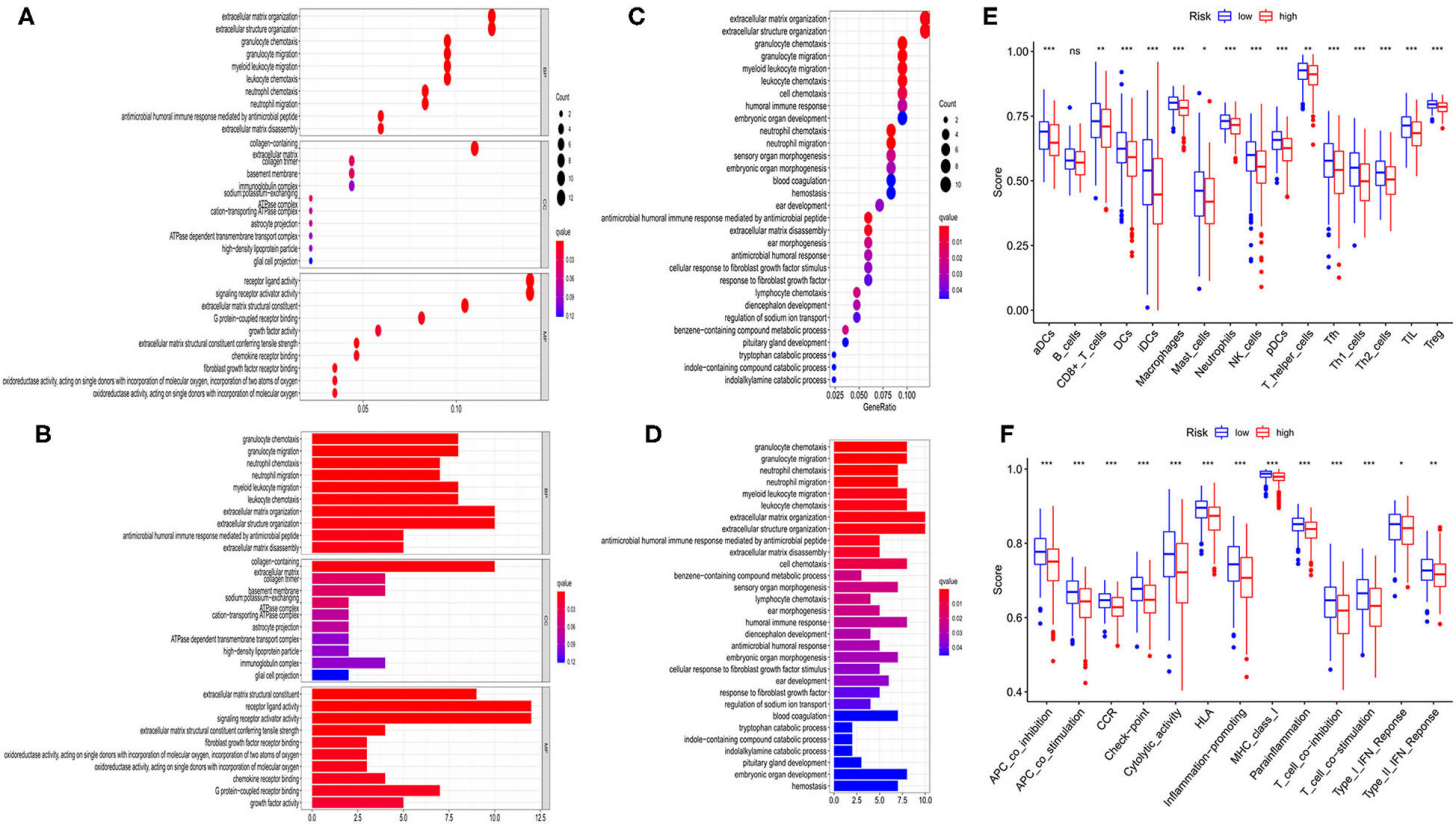
Functional analysis based on the DEGs between the two-risk group in TCGA cohort. **(A,B)** Bubble and barplot graph for GO enrichment. **(C,D)** Bubble and barplot graph for KEGG pathways. **(E)** Comparison of the infiltration of 16 immune cells between low- and high-risk group. **(F)** Comparison of the immune functions between low- and high-risk group.

According to the risk model, 374 OC patients were equally divided into low- and high-risk groups. The DEGs between the 2 groups were then screened out by using the criteria: FDR <0.05 and |log_2_FC| ≥ 1. To estimate the function of these DEGs, GO and KEGG pathway analyses were performed. As we could see, both GO (Figures 6A,B) and KEGG (Figures 6C,D) indicated that these DEGs were highly correlated with immune cell aggregation and immune response.

To further evaluate the relationship between risk group and immune cell infiltrations, we used ssGSEA to evaluate the immune cell features and the related pathways. As shown in Figure 6E, the high-risk group was found to be with a universally lower immune cells infiltration, particularly in antigen-presenting cells (aDCs, DCs, iDCs, and pDCs), macrophages, neutrophils, NK cells, T helper cells (Tfh, Th1, Th2), TIL, and Treg cell (all *P* < 0.001). Moreover, the functions of antigen presentation, immune activation, and immune surveillance were at lower levels in the high-risk group (Figure 6F). These results indicated that these ferroptosis-related genes may have strong correlations with the immune state of OC.

## Discussion

Overall, in this study, we demonstrated that the ferroptosis regulators were strongly connected with ovarian cancer. Firstly, we identified 57 DEGs between normal and tumor tissues, and based on these DEGs, OC could be well distinguished into 2 subtypes. We further combined the OS data and applied the LASSO regression model to set up a risk model, which was then proved to have a favorable predictive effect of prognosis in both TCGA and GEO cohorts. Functional analyses indicated the enrichment of the immune-related pathways, and then we found the lower immune-activation in high-risk OC cases.

In the past few decades, clinical features such as age, TNM stages, and some serum markers are commonly used to predict the prognosis of OC patients. However, due to individual differences, these single factors are not accurate enough and are less able to improve treatment options. With the rapid progression of gene sequencing technology, lots of genes' mRNA levels were found as predictive biomarkers in malignancies. However, a single gene is usually with low predictive effects as its expression level could be regulated by various signaling pathways. Applying the key regulators that function in the same signaling pathway to construct multi-gene-based models is essential to improve the predictive accuracy and could help to explore new targeted therapeutic methods. Recently, ferroptosis have been proved to be closely related to the development, chemoresistance, and occurrence of OC by large numbers of studies (Worley et al., 2019; Huang et al., 2020; Liu et al., 2020), however, the connections to the prognosis of OC patients were still unknown. Our studies presented a 5-ferroptosis-related signature (*ALOX12, ACACA, SLC7A11, FTH1*, and *CD44*) and found it could well predict the prognosis of OC patients. Moreover, the gene signature was proved to be better in predicting OS than age or tumor grade.

Among the 5 genes in the risk signature, *ALOX12* is known as a lipoxygenase, mainly to metabolize arachidonic acid to 12S-hydroperoxy eicosatetraenoic acid (12(S)-HpETE) (Zheng et al., 2020). In the procedures of ferroptosis, excessive accumulation of lipid peroxides leads to cell death, and these lipid peroxides can be induced by lipoxygenase production (Bayir et al., 2020). It has been reported that inactivation of *ALOX12* attenuated *p53*-mediated ferroptosis and abolished *p53*-dependent inhibition of tumor growth, indicating that *ALOX12* plays a key role in *p53*-mediated iron death (Chu et al., 2019). There's still no evidence whether *ALOX12* can affect the survival time of OC patients, but in other tumors, such as breast cancer(Huang et al., 2019) and colon cancer(Holzner et al., 2018), down-regulation of *ALOX12* could inhibit the invasive ability and enhance the chemosensitivity of tumor cells. We can infer that *ALOX12* may be associated with poor outcomes of OC patients, and we did find a higher expression of *ALOX12* in the high-risk group in this study.

*SLC7A11*, a crucial member participated in Xc- system, which is a cystine-glutamate reverse transporter process. Multiple cells rely on the Xc- system for cystine uptake, and this is a rate-limiting step in the synthesis of cysteine (Koppula et al., 2020). Blocking *SLC7A11* can lead to a decrease in intracellular cysteine, inhibit the lipid repair function of *GPX4*, and finally strengthen ferroptosis by affecting the redox state of the cell (Goji et al., 2017). Yin et al. (2019) found that lower expression of *SLC7A11* was associated with a shorter OS time of OC patients, and this conclusion was in accordance with ours that *SLC7A11* expressed in a lower level in the high-risk group. In addition, current studies indicated that *SLC7A11* mediates the chemosensitivity and chemoresistance of tumors and can be a potential target for improving chemotherapeutic responses (Huang et al., 2005).

Acetyl coenzyme A carboxylase (*ACACA*) is a biotin carboxylase that catalyzes the ATP-dependent condensation of acetyl coenzyme A and carbonates to form malonyl coenzyme and this reaction governs the rate of the first stage of fatty acid synthesis (Brownsey et al., 2006). When cells are stimulated by iron death, *AMPK* is activated, which in turn phosphorylates and inhibits the activity of the *AMPK* downstream substrate acetyl coenzyme A carboxylase 1, as well as inhibits fatty acid synthesis, thereby slowing the accumulation of lipid oxides and the onset of iron death (Li et al., 2020a). *ACACA* was proved to be a poor prognostic biomarker in various cancers (Chajès et al., 2006; Fang et al., 2014), and we also found it high-expressed in the high-risk group.

*CD44* is an extremely widely distributed cell surface transmembrane glycoprotein that is mainly involved in heterogeneous adhesion (Chen et al., 2018). The newest research indicated that *CD44* played important roles that could stabilize *SLC7A11* by increasing the recruitment of *OTUB1* and promotes the interaction of *SLC7A11* with *OTUB1*, thereby inhibiting the ferroptosis in tumor cells (Liu et al., 2019). A meta-analysis manifested that high expression of *CD44* was correlated with chemoresistance and shorter disease-free survival (DFS) time, but not affect OS in OC patients (Zhao et al., 2016). We found *CD44* was abundant in the low-risk subgroup and the specific role of *CD44* still needs further exploration.

*FTH1* (Ferritin heavy polypeptide 1) is a ubiquitous intracellular protein that stores iron. In the ferroptosis procedure, *FTH1* was a negative regulator in many cell lines (Chen et al., 2020; Tian et al., 2020). It has been reported that *NCOA4* could bind to *FTH1*, resulting in *FTH1* degradation, over-releasing iron ions, and ultimately leading to the development of ferroptosis (Zhang et al., 2018). Lobello et al. (2016) found that lower *FTH1* predicted a shorter survival time in OC patients, and knockout of FTH1 would enhance the viability and aggression in SKOV3 cells. In the high-risk group in our study, *FTH1* was also at a lower expression.

In summary, *ALOX12* and *ACACA* were known as the promoters of ferroptosis, while *SLC7A11, CD44*, and *FTH1* have the opposite function. Based on the expression profiles of these genes, we can infer that the process of ferroptosis is more active in the high-risk OC group. However, the current view indicated that promoting the course of ferroptosis will be always connected to cancer cell death thus prolongs the survival duration of patients. We all know that cell death is often accompanied by inflammatory responses, and so does in ferroptosis. In tissues with ferroptosis, immunofluorescence staining by *F4/80* showed significant activation of macrophages (Shah et al., 2018). However, our subsequent results showed that the immune cells including macrophages were significantly at a lower level in the high-risk group and we guess that the inverse in our study may be caused by the low sensitivity to ferroptosis of OCs in the high-risk group. Besides, both *ALOX12* and *ACACA* take part in the procedure of fat metabolism, and their expressions were observed to be significantly elevated in the high-risk group, indicating the active lipid metabolism may affect the sensitivity of OC cells to ferroptosis. But the exact mechanisms of how these five genes interact to affect iron metabolism and ferroptosis resistance is unclear, further studies are needed.

We also employed the GO and KEGG analysis and found these DEGs between the two- risk subgroups were strongly connected to immunity. However, the relationships between ferroptosis and tumor immunity have not been elucidated for years. Recently, a study manifested that IFN-γ released from CD8^+^ T cells could down-regulate the expression of cystine transport proteins on the tumor cell surface, thereby enhancing lipid peroxidation and iron death of tumor cells (Wang et al., 2019). That's to say, the tumor cell's sensitivity to ferroptosis is parallel to immune functions so that low immune cell infiltrations in the high-risk group indicated that less ferroptosis occurred. Besides, tumor cells undergoing ferroptosis will release arachidonic acid thereby act as immune activators to stimulate antitumor immunity (Friedmann Angeli et al., 2019), and according to this, we can also speculate that ferroptosis were inactive in the high-risk group. High-risk OCs were accompanied by the impairment of various immunities, therefore, exploring the specific mechanisms between ferroptosis and immunity is essential for improving the survival rate of OC patients.

## Conclusion

In summary, our study indicated that ferroptosis is correlated to the development and the progress of OC, since most of the ferroptosis-related genes were expressed differently between normal and OC tissues. We constructed and validated a gene signature associated with ferroptosis that can accurately predict the prognosis of OC patients. Moreover, we found the DEGs between the low- and high-risk subgroup divided by our risk model were associated with tumor immunity. Our study provides novel markers for evaluating OC prognosis and uncovers significant proofs for future research on the mechanisms between ferroptosis-related genes and the immunity of ovarian cancer.

## Data Availability Statement

The original contributions presented in the study are included in the article/Supplementary Material, further inquiries can be directed to the corresponding author/s.

## Author Contributions

HQ and YY made contributions to the conception of this study. YY and QD analyzed the data and wrote the manuscript. SL and JH helped with the analysis of the data and revised the manuscript. All authors contributed to the article and approved the submitted version.

## Conflict of Interest

The authors declare that the research was conducted in the absence of any commercial or financial relationships that could be construed as a potential conflict of interest.

## Supplementary Material

Supplementary Figure 1 Heatmap of the DEGs between 88 normal and 379 OC tissues.

Supplementary Figure 2 Heatmap and clinicopathologic characters of the 2 clusters classified by these ferroptosis-related DEGs (tumor grade: G1, High differentiated; G2, Moderate differentiated; G3, Poor differentiated).

Supplementary Figure 3 **(A)** LASSO regression of the 5 OS-related regulators. **(B)** Cross-validation for tuning the parameter selection in the LASSO regression.

## Abbreviations

OC: ovarian cancer
LASSO: least absolute shrinkage and selection operator
TCGA: The Cancer Genome Atlas database
GEO: Gene Expression Omnibus
GTEx: Genotype-Tissue Expression
GO: Gene Ontology
KEGG: Kyoto Encyclopedia of Genes and Genomes
DEGs: differentially expressed genes
PCA: Principal Component Analysis
t-SNE: t-distributed stochastic neighbor embedding
ssGSEA: single-sample gene set enrichment analysis.
