# Supplementary figures and images for: A Novel Defined Risk Signature of the Ferroptosis-Related Genes for Predicting the Prognosis of Ovarian Cancer
Source: Front Mol Biosci. 2021 Apr 1;8:645845. doi: 10.3389/fmolb.2021.645845 (PMC8047312; doi:10.3389/fmolb.2021.645845)

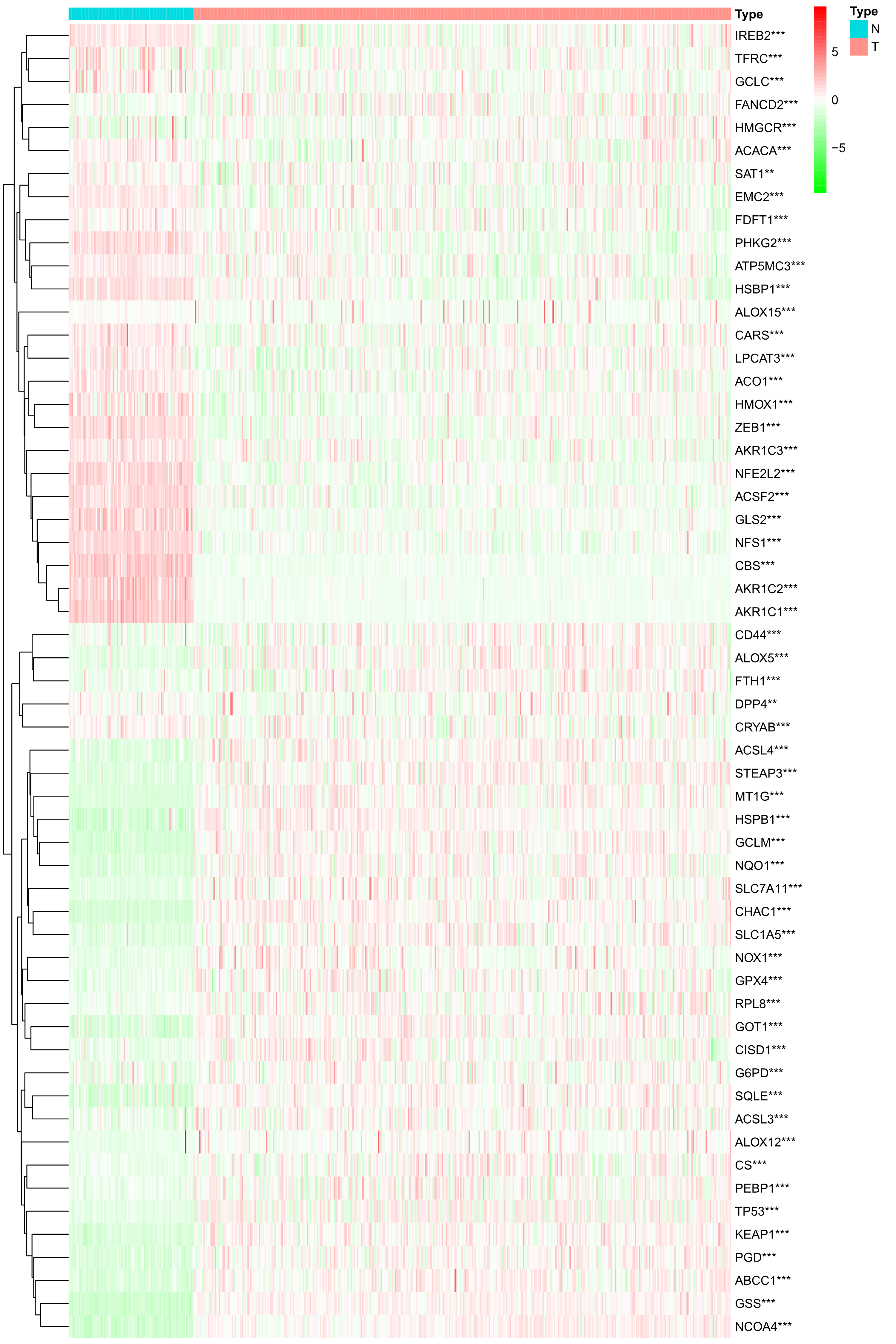

Supplement: Supplementary Figure 1 — Heatmap of the DEGs between 88 normal and 379 OC tissues. [file Image_1.TIF]

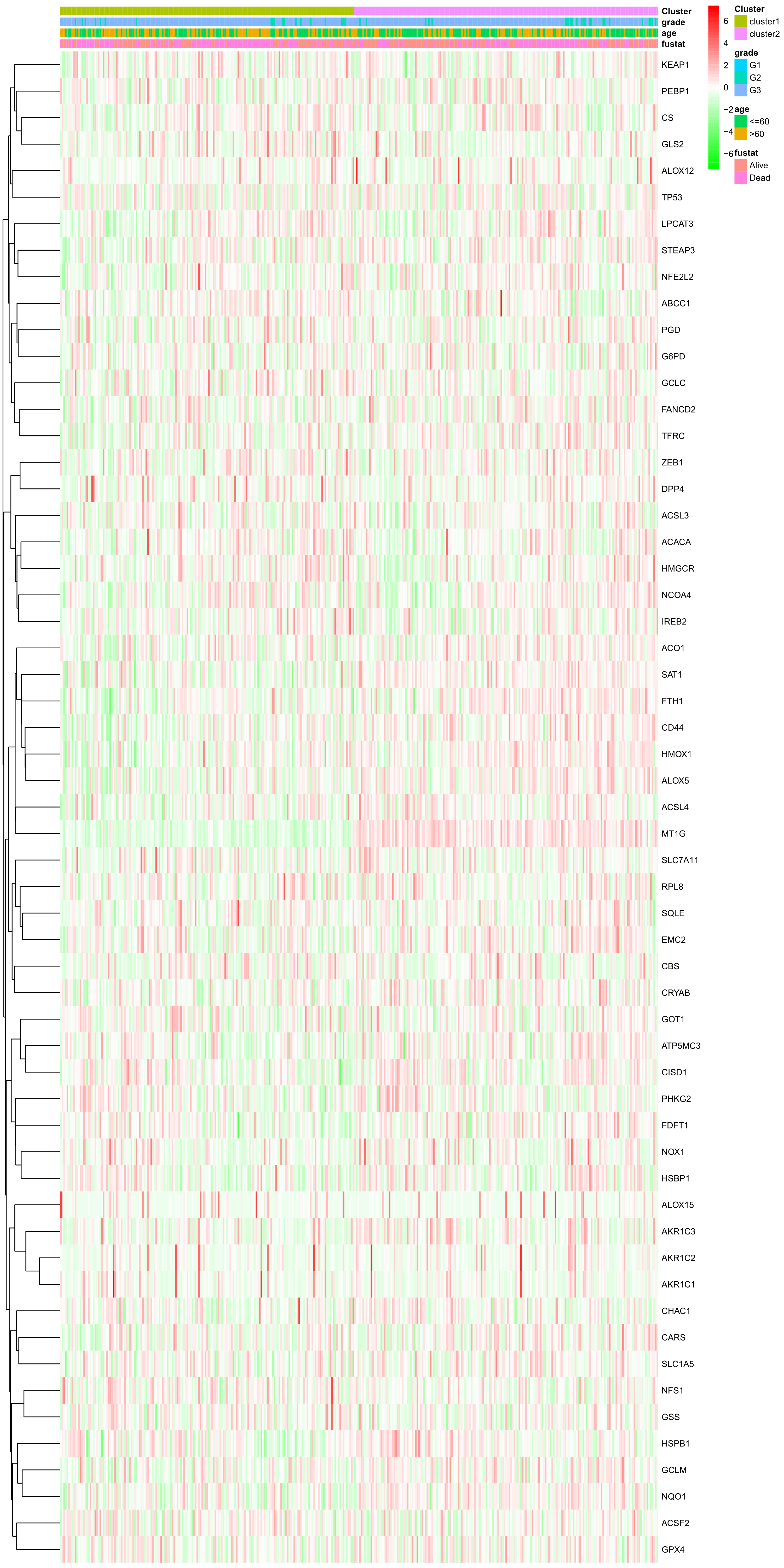

Supplement: Supplementary Figure 2 — Heatmap and clinicopathologic characters of the 2 clusters classified by these ferroptosis-related DEGs (tumor grade: G1, High differentiated; G2, Moderate differentiated; G3, Poor differentiated). [file Image_2.TIF]

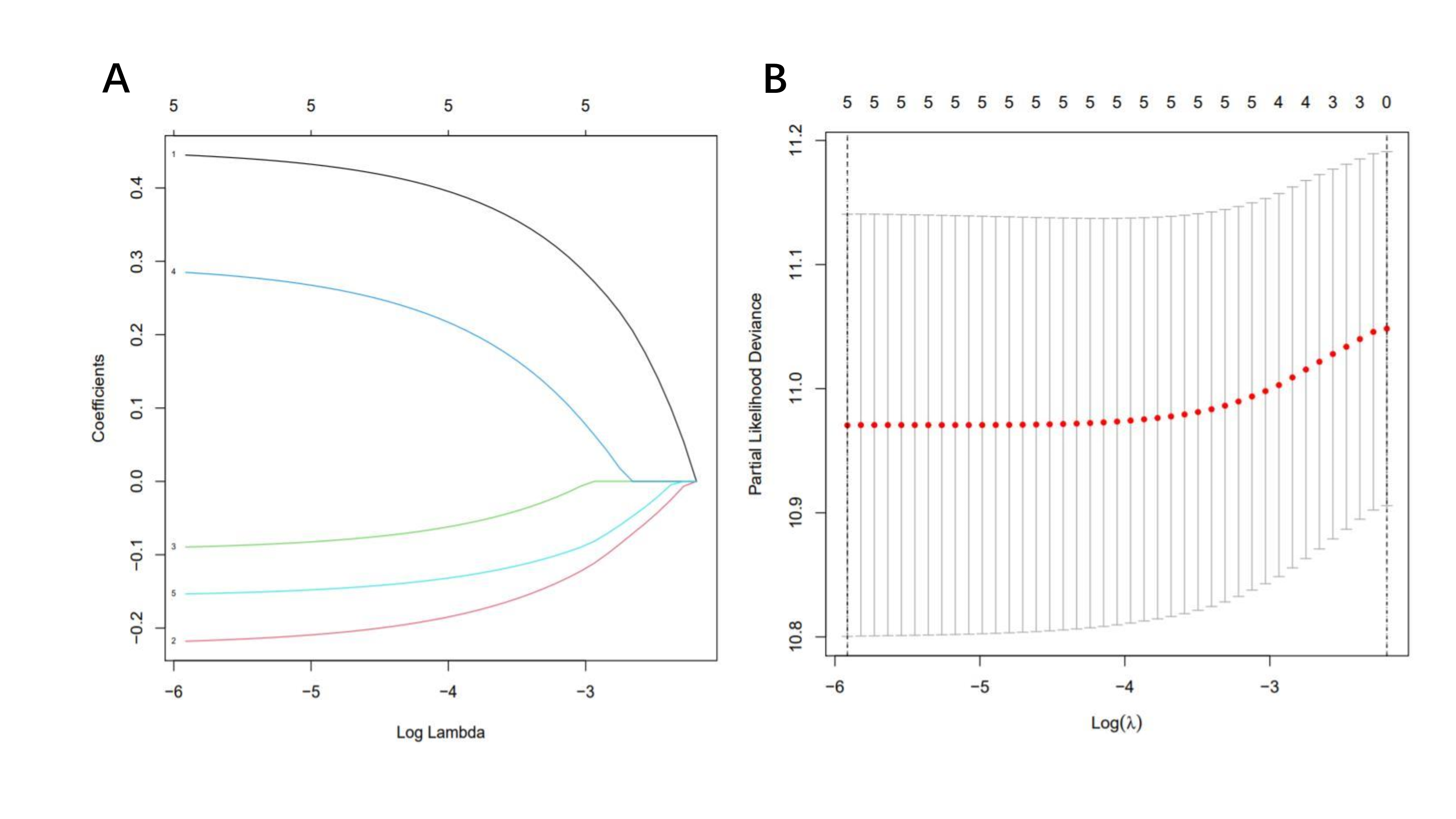

Supplement: Supplementary Figure 3 — (A) LASSO regression of the 5 OS-related regulators. (B) Cross-validation for tuning the parameter selection in the LASSO regression. [file Image_3.TIF]
